# Dynamic imaging of macrophages in MASLD: A major interest in insulin resistance and outside the liver

**DOI:** 10.1016/j.jhepr.2025.101352

**Published:** 2025-02-01

**Authors:** Nicolas Lanthier

**Affiliations:** 1 Service d’Hépato-Gastroentérologie, Cliniques universitaires Saint-Luc, UCLouvain, 1200 Brussels, Belgium; 2 Laboratory of Gastroenterology and Hepatology, Institut de Recherche Expérimentale et Clinique, UCLouvain, 1200 Brussels, Belgium

To the Editor:

Insulin resistance is a central pathophysiological feature of metabolic dysfunction-associated steatotic liver disease (MASLD) that is linked to its co-morbidities, such as visceral adiposity, type 2 diabetes, atherosclerosis and hepatocellular carcinoma (HCC). In this context, hepatic macrophages play a major role both in the pathogenesis of insulin resistance^1^ and MASLD.^2^ However, the subject is quite complex and hepatic macrophage populations may be considered beneficial or deleterious depending on their activation state.^3^ In particular, in the case of MASLD, resident macrophages are depleted while additional macrophage populations, including lipid-associated macrophages (LAMs), are recruited.^4^^,^^5^

In the recent issue of *JHEP Reports*, Bin Q. Yang and colleagues elegantly demonstrated that it was possible to monitor (by imaging) the loss of the CD163 marker, typical of resident liver macrophages, and the recruitment of CCR2-positive macrophages during the development of metabolic dysfunction-associated steatohepatitis (MASH),^6^ the severe inflammatory form of MASLD, which is associated with greater insulin resistance and is the target of current therapeutic trials.^4^ This was demonstrated in a choline-deficient L-amino acid defined (CDAA) diet-induced MASLD model, typical of MASH, by corroborating flow cytometry and immunofluorescence analyses with amazing dynamic *in vivo* PET data. The authors also showed that in the event of disease resolution, the CCR2 signal disappeared but, surprisingly, the low CD163 signal persisted.^6^ This is linked to the cessation of the toxic diet, but one can imagine studying the effect of other pharmacological or nutritional interventions with other models. The authors mention that their study does not enable assessment of the recruitment of other cell types which are also visible in MASH. However, experiments in animals and humans have clearly shown that liver macrophages are very rapidly activated (compared to other cell types) and that they play a pivotal initial role in the future development of the disease^2^ and associated insulin resistance.^7^

This exciting work gives rise to several perspectives. Although CDAA animals develop fibrosing MASH, they do not gain weight or develop adiposity or insulin resistance. In this sense, this model is not representative of the metabolic situation of MASLD in humans. However, the authors showed (by imaging) the same liver CCR2 uptake in an obesogenic diet mouse model.^6^ Importantly, we know that the recruitment of macrophages to visceral adipose tissue is also highly detrimental both in terms of insulin resistance and MASLD severity.^8^ It would be very interesting to study macrophagic recruitment by imaging jointly in the liver and the different white adipose tissues in the context of insulin resistance. This would enable us to understand the dynamics of macrophage recruitment in these organs under pathological conditions and according to therapeutic interventions. In the longer term, an increase in macrophages in skeletal muscle has also been detected in cases of obesity.^8^ In addition to adipose tissue, skeletal muscle appears to be another important organ to consider in the pathophysiology of MASH.^9^ Although some experiments show that these muscle macrophages do not express CCR2,^10^ if this type of *in vivo* tracing experiment enabled dynamic studies to be carried out not only in the liver but also in other organs/tissues, it would help improve our understanding of pathophysiological mechanisms, inter-organ communication and the response to interventions. Thus, it will be very important to investigate this by developing imaging approaches in extrahepatic tissues and metabolic models that more closely resemble the situation in humans.

## Financial support

NL has a mandate as Clinical researcher from the FNRS, Belgium.

## Conflict of interest

The author declares that the research was conducted in the absence of any commercial or financial relationships that could be construed as a potential conflict of interest.

Please refer to the accompanying ICMJE disclosure forms for further details.
